# Challenges and Strategies for Promoting Health Equity in Virtual Care: Protocol for a Scoping Review of Reviews

**DOI:** 10.2196/22847

**Published:** 2020-12-07

**Authors:** Jamie Keiko Fujioka, Suman Budhwani, Tyla Thomas-Jacques, Kristina De Vera, Priyanka Challa, Kaitlin Fuller, Sophie Hogeveen, Dara Gordon, Simone Shahid, Emily Seto, James Shaw

**Affiliations:** 1 Women's College Hospital Institute for Health Systems Solutions and Virtual Care Toronto, ON Canada; 2 Institute of Health Policy, Management and Evaluation University of Toronto Toronto, ON Canada; 3 University of Toronto Libraries University of Toronto Toronto, ON Canada; 4 Centre for Global eHealth Innovation University Health Network Toronto, ON Canada; 5 Joint Centre for Bioethics University of Toronto Toronto, ON Canada

**Keywords:** health equity, digital health, virtual care, telemedicine, scoping review, COVID-19, challenge, strategy

## Abstract

**Background:**

The rapid virtualization of health services during the COVID-19 pandemic has drawn increasing attention to the impact of virtual care technologies on health equity. In some circumstances, virtual care initiatives have been shown to increase health disparities, as individuals from underserved communities are less likely to benefit from such initiatives.

**Objective:**

The purpose of this paper is to describe a protocol for a scoping review of reviews that aims to map review-level evidence that describes challenges and strategies for promoting effective engagement with virtual care technologies among underserved communities.

**Methods:**

Our methodology was adapted from seminal scoping review guidelines provided by Arksey and O’Malley, Levac at al, Colquhoun et al, and the Joanna Briggs Institute. Our search strategy was developed for the following databases: MEDLINE (on Ovid), EMBASE (on Ovid), CINAHL (on EBSCO), Scopus, and Epistemonikos. Supplementary searches will include the use of Google Scholar and reference tracking. Each citation will be independently screened by 2 researchers at the title and abstract level, and full-text screening will be performed in accordance with our eligibility criteria. The eligibility criteria focused on the inclusion of methods-driven reviews (ie, systematic reviews, scoping reviews, meta-analyses, realist reviews, and critical interpretative syntheses) to enhance rigor and quality. Other inclusion criteria included a focus on virtual care services that facilitate bidirectional patient-provider communication (ie, video, telephone, and asynchronous messaging visits) for underserved populations (ie, those who experience social disadvantage due to race, age, income, and other factors related to the social determinants of health).

**Results:**

This scoping review of reviews will provide a broad overview of identified challenges associated with the accessibility of virtual health care services among underserved communities. In addition, strategies for improving the access to, uptake of, and engagement with virtual care technologies among underserved communities will be identified. The knowledge synthesized from this review will aid in developing and implementing virtual services that acknowledge the unique needs of populations who experience barriers to care and disproportionately worse health outcomes. The results will also inform gaps in current research.

**Conclusions:**

The rapid shift toward virtual health services has highlighted the urgent need to critically examine the intersection of virtual care and health equity. Although technology-driven innovations in health care generally aim to improve access, quality, and health outcomes, it is also possible for these innovations to produce intervention-generated inequities. Assessing current review-level evidence on the key challenges and strategies for improving the application of virtual care in underserved communities is imperative for ensuring that virtual care benefits all populations.

**International Registered Report Identifier (IRRID):**

PRR1-10.2196/22847

## Introduction

In response to the COVID-19 pandemic, virtual visits have rapidly transitioned from representing a small fraction of health care delivery to being the primary means of connecting patients and providers [1-4]. Virtual care is a broad term that encompasses diverse forms of health services that occur outside of traditional face-to-face clinical encounters, including telephone calls, video visits, secure messaging, and email consultations [5]. Recognizing the urgent need to maintain care while supporting physical distancing measures, government agencies and other stakeholders have enabled the use of virtual care, and in some cases health care payors have implemented reimbursement policies to facilitate the provider uptake of virtual care services [6]. Some jurisdictions have also permitted the use of nonencrypted technologies, such as FaceTime, Google Hangouts, and Skype, for patient care by waiving penalties for noncompliance with pre-existing privacy and security regulations (ie, the Health Insurance Portability and Accountability Act in the United States) [7]. This has resulted in the rapid implementation of virtual care [8]. The potential benefits of virtual care include reductions in emergency department visits, conserved health care resources, and improved access to care for some patients [4,6].

Many anticipate that several of the physical distancing measures that were implemented due to the COVID-19 pandemic may continue for the long term and that virtual care will continue to be ubiquitous in health service delivery [9]. However, given the immense speed at which health care services are being virtualized, a central concern is that considerations for health equity may be overlooked during the implementation of virtual care services [9,10]. In some circumstances, virtual care initiatives have been shown to increase health disparities, as individuals from underserved communities (ie, those who experience social disadvantage due to factors such as race, gender, income, or other social determinants of health) are less likely to benefit from such initiatives [9]. There are a variety of documented reasons for this, including the lack of language accessibility within certain apps, lack of cultural relevance, lack of skill or comfort in using certain technologies, and lack of access to the technical infrastructure (ie, internet-enabled devices and high-speed internet) required for engagement [11,12]. These issues must be considered so that the ongoing application of virtual care does not exacerbate existing health disparities between people with the highest access to health systems and people who are the most underserved by health systems.

Although several reviews have identified gaps in technology access, use, or literacy [11-14], few have systematically identified barriers specific to virtual health services among diverse underserved populations, and little is known about strategies for overcoming such barriers. In response to this perceived gap of knowledge, this scoping review of reviews will examine the range, nature, and extent of current research that explores virtual care among underserved communities. We refer to underserved communities as populations defined by social, economic, or geographic characteristics that can lead to their exclusion from mainstream social life, which in turn may directly or indirectly impact their ability to obtain high-quality care and achieve desired health outcomes [15]. The term “underserved” is often used interchangeably with the terms “vulnerable” or “marginalized” to describe populations who are at greater risk of poor health status and health care access. However, in contrast to the concepts of vulnerability or marginalization, our use of the term “underserved” is meant to call attention to prevailing systemic issues that result in unmet needs for those served by the health care system [15]. Our scoping review of reviews will synthesize review-level evidence on the challenges associated with the accessibility of virtual care services. In addition, we will identify strategies for improving the access to, uptake of, and engagement with virtual care among underserved communities. The knowledge synthesized from this review will aid in developing and implementing guidelines for virtual care services that are cognizant of diverse needs and sensitive to various differences in individual and group characteristics. The objective of this protocol paper is to describe the rationale, scope, and methods for conducting this scoping review of reviews.

## Methods

### Protocol Design

To inform our research question, we chose to conduct a scoping review of reviews to comprehensively, systematically, and feasibly map a large and diverse body of literature. Our approach was informed by guidance from the methodological frameworks created by Arksey and O’Malley [16], Levac et al [17]**,** and Colquhoun et al [18], and further refinements were made by referencing guidelines from the Joanna Briggs Institute [19]. The methodological steps are as follows: (1) identifying the research question; (2) identifying relevant studies; (3) study selection; (4) charting the data; and (5) collating, summarizing, and reporting the results [16-18]. In the absence of established guidelines on a scoping review of reviews, we also drew guidance from other peer-reviewed scoping reviews of reviews [20,21] to develop a methodologically rigorous approach.

Standardized reporting guidelines outline elements that should be included in research studies to enhance their transparency [22]. For this protocol, we used the Preferred Reporting Items for Systematic Reviews and Meta-Analyses literature search extension (PRISMA-S) to report on the search strategy [23]. Moreover, we will apply the Preferred Reporting Items for Systematic Reviews and Meta-Analyses Extension for Scoping Reviews (PRISMA-ScR) for reporting results [22]. This scoping review is registered in the National Collaborating Centre for Methods and Tools [24].

### Step 1: Identifying the Research Question

To establish our research questions, we first conducted an exploratory review of the literature on health equity in virtual care interventions. Following this review and extensive consultations with the members of our research team who had subject matter expertise, we made iterative improvements to clarify the concept and purpose of this study. After refining the study purpose, we established the following overarching research question: what challenges and strategies related to enabling the access to, uptake of, and engagement with virtual care for people from underserved communities have been documented in the literature?

The following subquestions will be used to further inform our investigation: (1) what is the review-level evidence regarding challenges that inhibit the access to, uptake of, and engagement with virtual care technologies among underserved communities; and (2) what is the review-level evidence regarding strategies for improving the access to, uptake of, and engagement with virtual care technologies among underserved communities?

### Step 2: Identifying Relevant Studies

In order to identify relevant studies that would inform our research questions, we first operationalized the following 2 key concepts within our study: virtual care and underserved populations. We then decided on the types of studies that would be the most relevant to include in the search strategy.

#### Operational Definition of Virtual Care

For the purposes of this scoping review, we defined virtual care as “any interaction between patients and/or members of their circle of care, occurring remotely, using any forms of communication or information technologies, with the aim of facilitating or maximizing the quality and effectiveness of patient care” [5]. Since the COVID-19 pandemic has increased the need for remote clinical interactions (ie, not in-person interactions), this review will focus on technologies that facilitate bidirectional patient-provider communication that fall under category 2.4.1 Consultations between remote client and healthcare provider, in the World Health Organization’s Classifications of Digital Health Interventions [25]. These include established technologies, such as telemedicine (eg, phone or video-based consultations), that have arisen over the past few decades, alongside newer communication platforms, such as asynchronous consultations via SMS text messaging, email, patient portals, and third-party apps.

#### Operational Definition of Underserved Community

We defined an underserved community as a group of people with increased susceptibility to health and health care disparities due to a combination of individual, environmental, and social factors that have been collectively defined as social determinants of health [15]. This operational definition was created using specific search terms within the search strategy. Our inclusion criteria focused specifically on older age, gender, racial or cultural identity, immigration and refugee status, socioeconomic status, homelessness, and rurality within the broader search criteria. This is in contrast to clinically at-risk populations with behavioral or biological factors that render them at risk for poor health outcomes; reviews that focus exclusively on these populations will not be included within this review.

#### Type of Reviews

Since our objective is to summarize a broad and diverse range of literature on virtual care, we will limit the inclusion of study types to the following methods-driven reviews: systematic reviews, meta-analyses, meta-syntheses, scoping reviews, realist reviews, and critical interpretive syntheses. Including studies with well-developed and established review methodologies will help reduce the risk of capturing poor quality studies. In addition, synthesizing review-level evidence will allow us to capture the vast amount of published literature on virtual care interventions in a logistically feasible manner [17].

#### Search Strategy Development

We developed comprehensive search strategies in collaboration with an academic librarian (KF) at the University of Toronto. These search strategies were developed for the following databases: Ovid MEDLINE: Epub Ahead of Print, In-Process and Other Non-Indexed Citations, Ovid MEDLINE Daily, and Ovid MEDLINE 1946-present; EMBASE (on Ovid), CINAHL (on EBSCO); Scopus; and Epistemonikos. The databases were selected based on subject area coverage and functionality. Additionally, guidelines provided by Goosen et al [26] and Bramer et al [27] were applied to inform the database selection. A date limit of 2005 to the present date was placed on the search to capture the most current literature on virtual care. The search strategies used a combination of text words, keywords, and subject headings for each concept that were relevant to our operational definitions of virtual care and underserved community. To retrieve reviews, a third concept was added to the MEDLINE, EMBASE, and CINAHL search strategies, and review filters were applied in Scopus and Epistemonikos. A draft search strategy for MEDLINE is provided in Multimedia Appendix 1. Citations were exported from the databases into EndNote X9 (Clarivate Analytics) for deduplication using a deduplication methodology adapted from Bramer et al [28]. Results were then imported into Covidence, a systematic review software program that supports the screening and management of citations by multiple reviewers [29].

#### Supplementary Searching

To supplement our search, we will use Google Scholar to identify relevant reviews that were not captured during the database searches. Key terms for each concept will be applied, and the first 3 pages of the search results will be reviewed. Potentially relevant items will be selected and deduplicated against our original set of search results and sent for screening. In addition, we will hand search the reference lists of included studies to identify any potentially relevant citations that may have been missed during the initial database search.

### Step 3: Study Selection

The inclusion and exclusion criteria for study selection (Textboxes 1 and 2) were developed iteratively by the research team based on the previously mentioned operational definitions and search strategy.

**Inclusion criteria for study selection.**

**Types of participants**

- Reviews of interventions that target or describe the impact of the intervention on an underserved population (ie, those who experience social disadvantage due to older age, gender identity, racial or cultural identity, immigration or refugee status, low income or low socioeconomic status, and rurality).

**Concept**

- Reviews on health care–focused technological interventions.
- Reviews focused on virtual care interventions, as defined in Section 2.4.1, Consultations between remote client and healthcare providers, in the World Health Organization’s Classification of Digital Health Interventions. These include telephone communication; video communication; asynchronous SMS text messaging; asynchronous email messaging; portals, apps, and other applications for bidirectional patient-provider communication; and remote monitoring tools that incorporate bidirectional communication functionality (ie, the tools listed previously).

**Context**

- All health system settings in high-income countries.

**Types of evidence**

- Methods-driven literature reviews, including systematic reviews, meta-analyses, scoping reviews, realist reviews, and critical interpretive syntheses.

**Exclusion criteria for study selection.**

**Types of participants**

- Reviews of interventions that target or describe the impact of the intervention on a general or clinical population instead of underserved populations as described within our inclusion criteria.

**Concept**

- Reviews of technological interventions that do not explicitly focus on bidirectional provider-patient communication (eg, patient portals that only focus on providing patients with access to their health information, remote monitoring tools without bidirectional patient-provider communication functionality, and provider-provider communication tools).

**Context**

- Studies focused on middle-income and low-income countries.

**Types of evidence**

- Reviews and knowledge syntheses that are not methods based.
- Primary research studies that use qualitative and quantitative methods.
- Opinion papers, commentaries, editorial reviews, and letters to the editor.
- Study protocols, dissertations, and conference abstracts/proceedings.

A screening guide, which was developed by 1 reviewer (SB) with feedback from the research team, will be used to determine if the inclusion and exclusion criteria have been met. In total, 5 researchers (JKF, KD, PC, SB, and TTJ) will independently pilot test the screening guide with a test sample of 200 abstracts. Results will be discussed, and revisions to the screening guide will be made as needed. An example of an included article and an excluded article will also be presented to the project team to ensure the appropriateness of included articles.

After establishing the screening guide and completing a pilot test, a 2-stage screening process will be implemented. First, all available titles and abstracts will be independently screened by 2 reviewers to determine the eligibility of articles for inclusion. Reviewers will meet regularly to discuss any challenges related to study selection and refine the inclusion and exclusion criteria as needed. Conflicts will be resolved by a third reviewer or through group discussion. The second stage of study selection will involve the examination of the full-text articles accepted in the first stage of study selection to determine their eligibility for inclusion. Any included full-text articles will be independently reviewed by 2 reviewers based on the inclusion and exclusion criteria. Once again, any conflicts between reviewers will be resolved by a third reviewer or through discussion with the research team. The study selection process will be summarized in a PRISMA (Preferred Reporting Items for Systematic Reviews and Meta-Analyses) flow diagram.

### Step 5: Collating, Summarizing, and Reporting the Results

#### Data Extraction

A data extraction form will be developed and pilot tested by 5 reviewers (JKF, KD, PC, SB, and TTJ). A draft version of this form is provided in Multimedia Appendix 2. The data extraction form will be pilot tested on 2 articles to test for the form’s consistency and comprehensiveness in capturing relevant data. Changes will be made through team discussion after comparing pilot test results.

Based on the studies used for developing the search strategy, the proposed fields for extraction include the following: (1) review identifiers (ie, authors, year of publication, review type, number of studies in the review, reported timeframe, place of publication); (2) the nature of the virtual care intervention(s) (ie, categorization and purpose of the technology); (3) setting and population (ie, the physical setting or geographical location of the intervention and demographic characteristics of the population); (4) the reported challenges for implementation, adoption, and engagement; (5) the reported strategies for improving implementation, adoption, and engagement; and (6) key study outcomes or conclusions.

#### Data Synthesis

A qualitative descriptive approach will be used to synthesize the data collected. The common characteristics of review articles will be identified to descriptively analyze the extent, nature, and distribution of included review articles. These characteristics include the review articles’ methodology, technologies described, target population(s), country/region of origin, and content. In keeping with established scoping review guidelines [16-18], the level or quality of evidence will not be formally appraised. An inductive and exploratory analysis of the findings will be conducted to identify emergent concepts and recurring patterns that can be extracted from the included studies. Major themes and subthemes arising from the review literature will be summarized with a focus on describing major barriers to virtual care technologies and strategies for improving engagement, in line with the research objectives. Themes will be iteratively developed over a series of meetings, during which the researchers will cluster the results into higher order categories. This will lead to a narrative summary of major findings. Potential gaps in providing virtual care technologies to underserved populations will also be identified based on our summary of the review literature.

## Results

This scoping review is currently in the study selection phase. Electronic database searches were completed in August 2020, yielding 9666 unique references. Following title and abstract screening, 9526 records were excluded based on the inclusion and exclusion criteria. As a next step, the resulting 140 references will undergo full-text review. Data synthesis will follow, and the authors anticipate that the results of this study will be submitted for publication in January 2021.

## Discussion

### Protocol Overview

Digital health interventions that are developed without context and without sensitivity to diverse needs can exacerbate pre-existing health disparities, thereby widening the gap between those with privilege and those without [9,11,30]. To our knowledge, this is the first scoping review to use review-level evidence to assess the unique considerations of implementing virtual care services for underserved communities. Although documented discrepancies in the access to and utilization of digital health interventions are abundant [11,31], a comprehensive study that maps key challenges and strategies for promoting health equity in virtual care has yet to be conducted. By identifying the predominant barriers and facilitators related to virtual care use among diverse populations, our findings will offer providers, health system leaders, and policy makers evidence-informed recommendations to enhance equity when introducing technology-driven innovations in health care. In addition, unlike traditional scoping reviews, this protocol outlines a methodological approach to conducting a scoping review of reviews that systematically maps and synthesizes review-level evidence.

### Limitations

A potential limitation of this study is the lack of quality assessment for included articles. Although a quality appraisal is not required in scoping reviews [16,18,19], we hope to improve the quality and rigor of our approach by limiting our search to reviews with well-established methodologies (ie, systematic reviews, meta-analyses, and scoping reviews). We recognize that our focus on review-level evidence may exclude relevant primary research studies. However, since the literature on digital health interventions and underserved populations is vast, our focus on reviews will allow us to feasibly obtain a succinct overview of the field and increase the heterogeneity and breadth of reported virtual care interventions. In addition, operationalizing the term “underserved community” in our search was challenging due to the diverse characteristics that encompass this broad categorization of people. Several in-depth discussions and a careful review of the health equity literature was performed to help inform our operational definition of “underserved community”. Although our operationalization of underserved communities could not encompass every population that faces systematic barriers to health care, we hope that our choice of search terms is purposefully broad enough to identify relevant considerations for improving future implementations of virtual care. Future research should be considered to capture any populations that our scoping review of reviews may have excluded due to feasibility constraints.

### Conclusions

The rapid virtualization of health services during the COVID-19 pandemic has highlighted the urgent need to critically examine the intersection of virtual care and health equity. Although technology-driven innovations in health care generally aim to improve access, quality, and health outcomes, it is also possible for these innovations to produce intervention-generated inequities by differentially benefiting those with more social and economic privilege than others [30]. Our protocol outlining a scoping review of reviews is a methodologically rigorous approach to mapping comprehensive health service research on key challenges and strategies for improving the application of virtual care in underserved communities. The results from our scoping review of reviews will provide valuable insight for the promotion of health equity in virtual care and will reveal current knowledge gaps in research.

## Appendix

Multimedia Appendix 1 Search strategy for Ovid MEDLINE: Epub Ahead of Print, In-Process & Other Non-Indexed Citations, Ovid MEDLINE Daily, and Ovid MEDLINE 1946-Present.

Multimedia Appendix 2 Draft data extraction form.

## Abbreviations

PRISMA: Preferred Reporting Items for Systematic Reviews and Meta-Analyses
PRISMA-S: Preferred Reporting Items for Systematic Reviews and Meta-Analyses literature Search extension
PRISMA-ScR: Preferred Reporting Items for Systematic Reviews and Meta-Analyses Extension for Scoping Reviews
