# Supplementary material for: Challenges and Strategies for Promoting Health Equity in Virtual Care: Protocol for a Scoping Review of Reviews
Source: JMIR Res Protoc. 2020 Dec 7;9(12):e22847. doi: 10.2196/22847 (PMC7721627; doi:10.2196/22847)
Supplement: Multimedia Appendix 1 [file resprot_v9i12e22847_app1.docx]

### Appendix 1: Search Strategy for Ovid MEDLINE: Epub Ahead of Print, In-Process & Other Non-Indexed Citations, Ovid MEDLINE® Daily and Ovid MEDLINE® 1946-Present

Date Run: June 20, 2020
Search Strategy:

| **#** | **Searches** | **Results** |
| --- | --- | --- |
| 1 | exp Telecommunications/ or internet-based intervention/ | 91461 |
| 2 | (remot* adj2 (consult* or interact* or diagnos* or monitor* or treat* or therap* or care)).tw,kw,kf. | 5854 |
| 3 | (telemonitor* or telemedicine* or telecommunication* or telehealth*).tw,kf. | 21131 |
| 4 | ((remote or online or video* or text message* or telephone* or phone or phones or email* or virtual* or technolog* or iphone* or smartphone* or mobile application* or mobile app* or teleconferenc* or messenger or whatsapp or skype or zoom or instant messag* or tablet* or e-mail* or asynchronous messag* or synchronous messag* or Videoconferenc* or hotline* or helpline* or call center*) adj3 (communicat* or engag* or discuss* or care or interact* or clinical guidance)).tw,kf,kw. and patient*.tw. | 11768 |
| 5 | ((remote or online or electronic* or video* or text message* or telephone* or phone* or email* or technolog* or virtual* or iphone* or smartphone* or mobile application* or mobile app* or teleconferenc* or messenger or whatsapp or skype or zoom or instant messag* or tablet* or e-mail* or asynchronous messag* or synchronous messag* or Videoconferenc* or hotline* or helpline* or call center*) adj3 (consult* or appointment* or meet or meeting* or visit*)).tw,kw,kf. or virtual tool*.tw,kf. | 8718 |
| 6 | ((virtual* or digital*) adj3 (healthcare or health care or health strategy)).tw,kw,kf. or (virtual care or virtual health).tw,kf. or (rapid* adj3 virtual*).tw,kf. | 1464 |
| 7 | ((online or digital* or virtual*) adj3 (doctor* or physician* or clinic or clinics or nurse or nurses or nursing or medicine or medical)).tw,kw,kf. | 5110 |
| 8 | (digital health or digital first).tw,kf. | 1853 |
| 9 | ("e health*" or ehealth* or evisit* or "e-visit*").tw,kf. | 6656 |
| 10 | (online adj3 (healthcare or health care)).tw,kf,kw. | 389 |
| 11 | ((virtual* or digital*) adj3 (healthcare or health care or health strategy)).tw,kf,kw. | 709 |
| 12 | (teleassist* or "tele‐assist*" or teleaudiolog* or "tele‐audiolog*" or telebased or "tele‐based" or telecancer or "tele‐cancer" or "tele‐cardiolo*" or telecardiolog* or teleconsult* or "tele‐consult*" or telecounselling or "tele‐counselling" or telecounseling or "tele‐counseling" or teledental or "tele‐dental" or telederm* or "tele‐derm*" or telediagnos* or "tele‐diagnos*" or teledialysis or "tele‐dialysis" or teleecho* or "tele‐echo*" or teleemerg* or "tele‐emerg*" or teleepileps* or "tele‐epileps*" or telefollow* or "tele‐follow*" or teleguidance or "tele‐guidance" or "tele‐health*" or telehome* or "tele‐home*" or teleICU or "tele‐ICU" or teleintervention* or "tele‐intervention*" or telemanag* or "tele‐manag*" or telemedicine or "tele‐medicine" or telemental* or "tele‐mental*" or telemonitor* or "tele‐monitor*" or telenurs* or "tele‐nurs*" or teleoncolo* or "tele‐oncolo*" or teleopthalm* or "tele‐opthalm*" or telepalliat* or "tele‐palliat*" or "tele‐patholog*" or teleprocedu* or "tele‐procedu*" or telepsych* or "tele‐psych*" or teleradiol* or "tele‐radiol*" or telerefer* or "tele‐refer*" or telerehab* or "tele‐rehab*" or telesurger* or "tele‐surger*" or telesurgic* or "tele‐surgic*" or teletherap* or "tele‐therap*" or teletreat* or "tele‐treat*" or teletriage or "tele‐triage").tw,kf. | 23364 |
| 13 | (tele adj (care or counselling or counseling or diagnos* or health* or intervention* or manag* or therap* or treat* or medicine or medical or nursing)).tw,kw. | 384 |
| 14 | ("e‐care" or ecare or "e‐consult*" or econsult* or "e‐diagnos*" or ediagnosis* or "e‐medicine" or emedicine or "e‐nurse*" or enurse* or "e‐nursing" or enursing or "e‐physician*" or ephysician* or "e‐psych*" or epsych* or "e‐therapy" or etherapy or mhEALTH or "M‐HEALTH").tw,kf. | 4823 |
| 15 | ((online or video* or text message* or telephone* or phon* or email* or virtual* or technolog* or iphone* or smartphone* or mobile application* or mobile app* or teleconferenc* or messenger or instant messag* or whatsapp or skype or zoom or tablet* or e-mail* or asynchronous messag* or synchronous messag* or Videoconferenc* or hotline* or helpline* or call center*) adj2 (care or counselling or Counseling or diagnos* or health* or intervention* or manag* or therap* or treat* or medicine or medical or nursing)).tw,kw,kf. | 63528 |
| 16 | (virtual* adj3 monitor*).tw,kw,kf. | 139 |
| 17 | ((implant* sensor* or body sensor*) adj4 (diagnost* or monitor* or report*)).tw,kf. | 70 |
| 18 | mobile health monitor*.tw,kf. | 37 |
| 19 | computers, handheld/ or smartphone/ | 7796 |
| 20 | Mobile Applications/ | 5800 |
| 21 | exp Wearable Electronic Devices/ | 11582 |
| 22 | Computers/ or exp Microcomputers/ or Minicomputers/ | 69847 |
| 23 | internet/ or internet access/ or "internet of things"/ | 72614 |
| 24 | or/1-23 [telemedicine 1] | 316750 |
| 25 | Health Equity/ or (health adj3 (equit* or disparit*)).tw,kf. or Health Status Disparities/ | 34265 |
| 26 | communication barriers/ or digital divide/ or limited english proficiency/ | 6597 |
| 27 | (((language* or communicat*) adj3 (barrier* or challeng*)) or limited english proficiency).tw,kf. | 6921 |
| 28 | exp culture/ or (cultur* adj3 (communit* or person* or population* or disparit* or inequalit* or equity or factor* or ident*)).tw,kf,kw. | 192237 |
| 29 | Minority Groups/ or Minority Health/ | 14302 |
| 30 | (minorit* adj3 (group* or population* or communit* or people* or person* or patient* or health)).tw,kf,kw. | 23943 |
| 31 | Race Factors/ | 178 |
| 32 | exp Continental Population Groups/ or exp Ethnic Groups/ | 293023 |
| 33 | (ethnic* adj3 (group* or communit* or person* or patient* or population* or disparit* or inequalit* or equity or ident*)).tw,kw,kf. | 59202 |
| 34 | ((racial* or race or amish or arab* or hispanic* or mexican* or jewish or roma or black* or asia* or Filipino* or russian* or indian* or chinese or ukrain* or korean or japanese or polish or south american* or african* or latino* or latina* or Anabaptist* or Anglican* or Apostolic* or Bahai* or Baptist* or buddhis* or hindu* or islam* or judaism or muslim* or catholicism or eastern orthodox* or Jehovah's Witness* or christian* or mormon* or Sikh* or Confucianism or Lutheran* or Mennonite* or Hutterite* or Mysticism* or Pentacostal* or Presbyterian* or Protestant* or Seventh Day Adventist* or Shinto* or monotheis*) adj3 (group* or communit* or person* or patient* or population* or disparit* or inequalit* or equity or factor* or ident*)).tw,kw,kf. | 242845 |
| 35 | exp Socioeconomic Factors/ or "Social Determinants of Health"/ | 449665 |
| 36 | exp religion/ or exp Spiritual Therapies/ | 68936 |
| 37 | sociological factors/ | 555 |
| 38 | (socioeconomic adj2 (factor* or disadvantag*)).tw,kw,kf. | 21342 |
| 39 | "social determinants of health".tw,kf. | 5054 |
| 40 | (marital status or education status or salaries or salary).tw,kf. | 29253 |
| 41 | (low income or working poor or poverty or precarious work or homeless* or income or employment or employed or economic status or salar* or pension* or career mobility or ghetto* or food insecurit*).tw,kf. | 555932 |
| 42 | (street* adj2 (people* or person* or individual* or youth* or population* or child* or men or women or man or woman)).tw,kf. | 1100 |
| 43 | ((vulnerabl* or transient*) adj2 (people* or person* or individual* or child* or youth* or population* or worker* or men or women or man or woman or communit*)).tw,kf. | 20113 |
| 44 | ("lack of housing" or substandard housing or unstabl* house* or underhoused or under housed or squatter*).tw,kf. | 691 |
| 45 | homeless persons/ or homeless youth/ | 8868 |
| 46 | Working Poor/ | 15 |
| 47 | exp social discrimination/ or social marginalization/ or social stigma/ | 14414 |
| 48 | (social adj3 (class or mobilit* or margin* or status or condition* or exclusion* or inequit* or equity or discriminat* or segretat*)).tw,kw. or social capital.tw,kf. | 34435 |
| 49 | Vulnerable Populations/ | 10424 |
| 50 | ((vulnerabl* or underserv* or disadvantag* or uninsured) adj3 (group* or population* or communit* or people* or person* or youth* or patient* or child* or men or man or woman or women)).tw,kw,kf. | 46060 |
| 51 | Refugees/ | 10122 |
| 52 | (refugee* or Asylum Seeker* or displaced person* or asylee*).tw,kf. | 11865 |
| 53 | "Emigration and Immigration"/ | 25126 |
| 54 | "Transients and Migrants"/ | 11542 |
| 55 | (immigrant* or immigration* or emigrant* or emigration*).tw,kf. | 37780 |
| 56 | (migrant* or incomer* or "in comer*" or "new comer*" or newcomer*).tw,kf. | 21136 |
| 57 | Prisoners/ | 16488 |
| 58 | Criminals/ | 4880 |
| 59 | (inmate* or jail or prisoner* or criminal*).tw,kf. | 35348 |
| 60 | Sex Workers/ or Sex Work/ | 7589 |
| 61 | (prostitut* or sex worker*).tw,kf. | 9079 |
| 62 | Drug Users/ or Needle Sharing/ | 4644 |
| 63 | exp Substance-Related Disorders/ or exp Drug Misuse/ | 278222 |
| 64 | exp Cocaine/ or exp Analgesics, Opioid/ or exp Benzodiazepines/ or exp Illicit Drugs/ | 207156 |
| 65 | Behavior, Addictive/ | 9792 |
| 66 | (drug abuse or alcoholism or "substance use disorder*" or alcoholic* or drug misuse or drug overuse or drug user* or drug addict*).tw,kf. | 143659 |
| 67 | (drug* adj3 (dependen* or abuses or abusing or disorder*)).tw,kf,kw. | 20014 |
| 68 | ((non prescription drug* or nonprescription drug* or illegal drug* or street drug* or Illicit drug*) adj3 (user$1 or abuse or abusing or abuses or addict* or misus* or dependen* or disorder*)).tw,kf,kw. or ((intravenous* or inhalant*) adj1 (abuse or abusing or abuses or misus* or disorder*)).tw,kf. | 2537 |
| 69 | ((heroin* or analgesic* or opiate* or opioid* or morphin* or morfin* or methadone* or oxycodone* or oxycontin* or narcotic* or hydrocodone* or hydromorphone* or codeine* or fentanyl* or meperidine* or oxymorphone* or propoxyphene* or tramadol* or buprenorphine* or marijuana* or cannabis* or cocaine* or ketamine* or amphetamine* or Methamphetamine* or phencyclidine* or benzodiaz* or alcohol*) adj3 (user$1 or abuse or abusing or abuses or addict* or misus* or dependen* or disorder*)).tw,kf,kw. | 97727 |
| 70 | Health Services for Persons with Disabilities/ | 108 |
| 71 | exp Disabled Persons/ or exp Intellectual Disability/ | 156902 |
| 72 | exp Hearing Disorders/ | 86647 |
| 73 | exp Vision Disorders/ | 71698 |
| 74 | (vision* adj3 (impair* or disorder*)).tw,kw,kf. | 5362 |
| 75 | ((hear* or visual) adj3 (impair* or disorder*)).tw,kw,kf. | 41484 |
| 76 | (Hearing Loss or Deaf* or tinnitus or hyperacusis or low vision or blind or blindness or Scotoma or Diplopia or keratomalacia or xerophthalmia*).tw,kf. | 312851 |
| 77 | ((ear adj1 (buzz* or ring*)) or (blurry adj1 vision*)).tw,kf. | 546 |
| 78 | ((disabl* or impair*) adj3 (people* or person* or individual* or child* or youth* or population* or worker* or men or women or man or woman or communit* or physical*)).tw,kw,kf. | 42607 |
| 79 | Rural Health/ | 23349 |
| 80 | exp Rural Health Services/ | 12833 |
| 81 | Rural Population/ | 58942 |
| 82 | ((rural or remote) adj3 (group* or population* or communit* or people* or youth* or child* or men or man or women or woman or person* or patient* or equity or disparit* or inequalit* or equity)).tw,kw,kf. | 52952 |
| 83 | exp disorders of sex development/ | 33057 |
| 84 | Health Services for Transgender Persons/ | 133 |
| 85 | exp Sexuality/ | 41445 |
| 86 | exp "Sexual and Gender Minorities"/ | 6163 |
| 87 | exp Gender Identity/ | 19575 |
| 88 | Gender Dysphoria/ | 468 |
| 89 | ((gender* or sexual*) adj3 (fluid* or minorit* or identit* or diverse or confirmation or non conform* or surg* or reassignment or change or disorder* or group* or population* or communit* or people* or person* or patient* or orientation)).tw,kw,kf. | 67419 |
| 90 | (bicurious or bisexual or bisexuality or bisexuals or "cross sex" or crossgender or F2M or "female-to-male" or gay or gays or "gender change" or "gender dysphoria" or "gender identity" or "gender queer" or "gender reassign" or "gender transform" or "gender transition" or genderqueer or GLB or GLBQ or GLBs or GLBT or GLBTQ or heteroflexible or homosexual or homosexualities or homosexuality or homosexuals or intersex or lesbian or lesbianism or lesbians or lesbigay or LGB or LGBQ or LGBS or LGBT or M2F or "male-to-female" or "men who have sex with men" or msm or queer or "same gender loving" or "same sex attracted" or "same sex couple" or "same sex couples" or "same sex relations" or "sex change" or "sex reassign" or "sex reversal" or "sex transform" or "sex transition" or "sexual and gender minorities" or "sexual and gender minority" or "sexual identity" or "sexual minorities" or "sexual minority" or "sexual orientation" or "sexual preference" or "trans female" or "trans male" or "trans man" or "trans men" or "trans people" or "trans person" or "trans woman" or "trans-sexuality" or transexual or transgender or transgendered or transgenders or transsexual or transsexualism or transsexuality or transsexuals or transvestite or "women loving women" or "women who have sex with women" or WSW).tw,kf. | 182978 |
| 91 | exp Aged/ | 3104139 |
| 92 | Geriatrics/ or Geriatric Nursing/ | 43023 |
| 93 | Health Services for the Aged/ | 17659 |
| 94 | (geriatr* or aged or elderly or elders or old age or gerontol* or centenarian* or nonagenarian* or octogenarian* or septuagenarian* or sexagenarian* or overaged or "oldest old").tw,kf. | 844248 |
| 95 | ((older or old) adj3 (person* or people* or population* or adult*)).tw,kf. | 146858 |
| 96 | (seniors not "high school").tw. | 6820 |
| 97 | Veterans/ | 16733 |
| 98 | Veterans Health/ | 1302 |
| 99 | Veteran*.tw,kf. | 36152 |
| 100 | Health Services, Indigenous/ | 3258 |
| 101 | (Athapaskan or Saulteaux or Wakashan or Cree or Dene or Inuit or Inuk or Inuvialuit* or Haida or Ktunaxa or Tsimshian or Gitsxan or Nisga'a or Haisla or Heiltsuk or Oweenkeno or Kwakwaka'wakw or Nuu chah nulth or Tsilhqot'in or Dakelh or Wet'suwet'en or Sekani or Dunne-za or Dene or Tahltan or Kaska or Tagish or Tutchone or Nuxalk or Salish or Stl'atlimc or Nlaka'pamux or Okanagan or Sec wepmc or Tlingit or Anishinaabe or Blackfoot or Nakoda or Tasttine or Tsuu T'inia or Gwich'in or Han or Tagish or Tutchone or Algonquin or Nipissing or Ojibwa or Potawatomi or Innu or Maliseet or Mi'kmaq or Micmac or Passamaquoddy or Haudenosaunee or Cayuga or Mohawk or Oneida or Onodaga or Seneca or Tuscarora or Wyandot or Aboriginal* or Indigenous* or Metis or red road or "on reserve" or off-reserve or First Nation or First Nations or Amerindian).mp. | 64577 |
| 102 | residential school*.mp. | 355 |
| 103 | autochtone*.mp. | 411 |
| 104 | first people*.mp. | 415 |
| 105 | (Native* adj1 (American* or man or men or women or woman or boy* or girl* or adolescent* or youth or youths or person* or adult or people* or Indian* or Nation or tribe* or tribal or band or bands)).tw,kf,kw. | 5861 |
| 106 | Alaska Native*.tw,kf. | 2747 |
| 107 | turtle island.tw,kf. | 7 |
| 108 | (urban adj3 (Indian* or Native*)).tw,kf,kw. | 1117 |
| 109 | (indian* adj3 (north americ* or american*)).tw,kw,kf. | 7710 |
| 110 | or/25-109 [population] | 6026001 |
| 111 | exp Technology Assessment, Biomedical/ | 11058 |
| 112 | meta-analysis/ or "systematic review"/ or meta-analysis as topic/ or network meta-analysis/ or exp "Review Literature as Topic"/ | 218860 |
| 113 | (meta analy* or metaanaly* or metasynthe* or meta-synthe* or knowledge synthesis or knowledge syntheses or meta regression* or metaregression*).tw,kf. | 178572 |
| 114 | (rapid adj2 (review$1 or overview$1)).tw,kf. | 1246 |
| 115 | (evidence adj2 (map or mapping or synthesis*)).tw,kf,kw. | 6832 |
| 116 | ((critical or interpretive) adj2 synthesis*).tw,kf,kw. | 592 |
| 117 | "review of reviews".tw,kf. | 554 |
| 118 | (systematic adj1 assess*).tw,kf. | 3004 |
| 119 | (research adj2 synthesis*).tw,kf. | 1278 |
| 120 | ((state-of-the-art or umbrella or evidence) adj2 (review* or overview* or assessment*)).tw,kf,kw. | 36962 |
| 121 | (methodologic* adj3 (review* or overview*)).tw,kf,kw. | 3788 |
| 122 | ((quantitative adj3 (review* or overview* or synthes*)) or (research adj3 (integrati* or overview*))).tw,kf,kw. | 10926 |
| 123 | (cochrane or (health adj2 technology assessment) or evidence report).jw. | 19505 |
| 124 | ((systematic or scoping or integrative or collaborative or realist or critical or interpretive) adj3 (review$1 or overview$1)).tw,kf,kw. | 211295 |
| 125 | Systematized Review*.tw. | 61 |
| 126 | Framework Synthesis*.tw. | 172 |
| 127 | Meta-Aggregation*.tw. | 105 |
| 128 | Qualitative Meta-Summar*.tw. | 12 |
| 129 | Scoping Stud*.tw,kf. | 354 |
| 130 | Systematic Map$1.tw,kf. | 66 |
| 131 | Comparative Effectiveness Review*.tw. | 183 |
| 132 | Mixed Methods Review*.tw. | 72 |
| 133 | Realist Synthesis*.tw. | 211 |
| 134 | (Cochrane or Campbell Collaboration or Joanna Briggs or Arksy or PRISMA* or RAMESES or MOOSE).tw. | 98338 |
| 135 | Concept Synthesis*.tw. | 31 |
| 136 | (CADTH or EEI-Centre Review or AHRQ or "Agency for Healthcare Research and Quality" or "Canadian Agency for Drugs and Technologies in Health" or "Centre for Reviews and Dissemination" or EUnethta or "European Network for Health Technology Assessment" or "Health Technology Assessment International" or HTAi or "National Collaborating Centre for Methods and Tools").tw. | 3405 |
| 137 | or/111-136 | 416561 |
| 138 | "Systematic Search and Review".tw. | 110 |
| 139 | (search method$1 or study selection or study screen* or screen* stud* or citation* screen* or screen* citation*).ab. | 29710 |
| 140 | (medline or pubmed or embase or psychlit or psyclit or psychinfo or psycinfo or cinahl or cinhal or science citation index or scopus or "web of science" or reference list* or bibliograph* or hand-search* or handsearch* or relevant journal* or manual search* or Covidence or DistillerSR or Rayyan or prospero or deduplication or de-duplication or reference manager* or endnote or refworks or zotero or mendeley or prisma or joanna briggs or revman or EPPI-Reviewer or SysRev).ab. | 248166 |
| 141 | (selection criteria or eligibility criteria or screening criteria or inclusion criteria or exclusion criteria).ab. | 147327 |
| 142 | (mantel haenszel or peto or der simonian or dersimonian or fixed effect* or latin square* or outcomes research or relative effectiveness).ab. | 32487 |
| 143 | (data collection or data analysis or data sources or data synthesis or data extraction).ab. | 179789 |
| 144 | (comparative adj3 (efficacy or effectiveness)).tw. | 12940 |
| 145 | (pool* adj3 analy*).ab. | 20112 |
| 146 | or/138-145 [methods] | 524074 |
| 147 | exp "review"/ or (literature adj3 (review$1 or overview$1)).tw. | 2774447 |
| 148 | 146 and 147 [methods and review] | 190835 |
| 149 | 137 or 148 [review filter] | 484252 |
| 150 | 24 and 110 and 149 | 4042 |
| 151 | (2005* or 2006* or 2007* or 2008* or 2009* or 2010* or 2011* or 2012* or 2013* or 2014* or 2015* or 2016* or 2017* or 2018* or 2019* or 2020*).dt,ez,da,dp. | 15833687 |
| 152 | 150 and 151 | 3713 |
