# Supplementary material for: Challenges and Strategies for Promoting Health Equity in Virtual Care: Protocol for a Scoping Review of Reviews
Source: JMIR Res Protoc. 2020 Dec 7;9(12):e22847. doi: 10.2196/22847 (PMC7721627; doi:10.2196/22847)
Supplement: Multimedia Appendix 2 [file resprot_v9i12e22847_app2.docx]

### Appendix 2: Draft Data Extraction Form

General

| Extractor Name/ID |  |
| --- | --- |
| Title of Article |  |
| Author(s) |  |
| Publication Year |  |

Methods

|  | Descriptions as stated in text |
| --- | --- |
| Review Type | Critical interpretive synthesis review  Meta-analysis review  Meta-synthesis review  Realist review  Scoping review  Systematic review |
| Review Objectives |  |
| Inclusion Criteria |  |
| Databases Searched |  |
| Date Range |  |
| Number of Studies Included |  |

Virtual Care

|  | Descriptions as stated in text |
| --- | --- |
| Description of technology used |  |
| Categorization of technology | Telephone communication  Video communication  Text messaging (asynchronous)  Email messaging (asynchronous)  Patient portals, app etc. with virtual care functionality |
| Notes |  |

Population

|  | Descriptions as stated in text |
| --- | --- |
| Describe characteristics of study population (i.e. what makes group underserved) |  |
| Notes |  |

Equity

| Is Equity explicitly stated? (Y/N) | Yes  No |
| --- | --- |

Findings: Strategies

| Access to virtual care |  |
| --- | --- |
| Uptake of virtual care |  |
| Engagement with virtual care |  |
| Notes |  |

Findings: Challenges

| Access to virtual care |  |
| --- | --- |
| Uptake of virtual care |  |
| Engagement with virtual care |  |
| Notes |  |

Other

|  | Description as stated in text |
| --- | --- |
| Other Outcomes (efficacy, feasibility, acceptability) |  |
| Key Conclusions of Study Authors |  |
| Notes |  |
